# HOW WELL DO INSTITUTIONALIZED ASSUMPTIONS ABOUT UNPAID CAREGIVERS MATCH THE EXPERIENCES OF HOME CARE CLIENTS?

**DOI:** 10.1093/geroni/igae098.1040

**Published:** 2024-12-31

**Authors:** Laura Funk, Kaitlyn Kuryk, Lauren Spring, Janice Keefe

**Affiliations:** University of Manitoba, Winnipeg, Manitoba, Canada; University of Manitoba, Winnipeg, Manitoba, Canada; Mount Saint Vincent University, Halifax, Nova Scotia, Canada; Mount Saint Vincent University, Halifax, Nova Scotia, Canada

## Abstract

Despite efforts to acknowledge diversity among unpaid caregivers, research, advocacy, practice and policy tends to be based in, and to reproduce, normative expectations and assumptions about who provides unpaid care and what this support looks like. The objective of this analysis is to generate unique insights into unpaid caregiving by exploring the situations of older adults receiving publicly funded home care and their experiences of accessing unpaid support. The analysis draws on case study interviews with twelve home care clients, their unpaid caregiver, home care aide and case coordinator, conducted at three points in time, before and after the Covid-19 pandemic (129 interviews). Analysis indicated only one participant had access to a caregiver that aligned with normative conceptualizations. Rather, situations arose in which: a) family members grappled with physical or mental health challenges limiting their participation in care (sometimes meaning the client is themselves a caregiver, or the caregiver is also receiving home care services); b) caregivers facing burnout sought to delimit their participation in care; c) family members’ participation was limited by older adults’ reluctance to accept their help; d) caregivers were largely unavailable, unreliable, or peripheral; or e) client’s unpaid support networks were diffuse without a clearly identifiable ‘caregiver.’ Findings are used to nuance and problematize normative assumptions about caregivers. Discussion highlights the need for research, policy and programs related to unpaid caregiving to better reflect the lived realities of unpaid support for older adults and often overlooked sources of diversity in caregiver circumstances and roles.

